# Is endobronchial ultrasound-guided transbronchial needle aspiration with a stylet necessary for lymph node screening in lung cancer patients?

**DOI:** 10.1590/1414-431X20176372

**Published:** 2017-08-17

**Authors:** Y. Xu, J. Lin, Y. Jin, X. Wu, H. Zheng, J. Feng

**Affiliations:** 1 Department of Respiratory Medicine, Tai Zhou Hospital of Zhejiang Province, LinHai, Zhejiang Province, China; 2 Department of Medical Record Library, Tai Zhou Hospital of Zhejiang Province, LinHai, Zhejiang Province, China; 3 Department of Pathology, Tai Zhou Hospital of Zhejiang Province, LinHai, Zhejiang Province, China

**Keywords:** Endobronchial ultrasound-guided, Transbronchial needle aspiration, Stylet, Cytology, Pathological diagnosis

## Abstract

During endobronchial ultrasound-guided transbronchial needle aspiration (EBUS-TBNA), a needle is commonly used with a stylet, although recently the stylet has been omitted. This prospective study aimed to compare the quality of specimens obtained by EBUS-TBNA performed with and without a stylet. Between November 2013 and November 2014, 131 patients with lung cancer underwent EBUS-TBNA, with a total of 148 mediastinal or hilar lymph nodes sampled both with and without an inner-stylet, yielding 296 cytological specimens. Specimens were scored cytologically using five parameters: background blood or clot, amount of cellular material, degree of cellular degeneration, degree of cellular trauma, and retention of appropriate architecture. The procedure with a stylet required significantly longer operation time than without a stylet (14.5±0.8 *vs* 12.7±1.1 min, P<0.001). Excellent specimens were obtained in 261/296 and 260/296 samples in the procedures with and without a stylet, respectively (P=0.9), while the remaining 35 and 36 samples, respectively, were adequate. The diagnosing and staging of lung cancer using EBUS-TBNA did not differ significantly between the groups. In conclusion, specimen collection by EBUS-TBNA without a stylet is easier and faster than the procedure using a stylet and absence of a stylet did not alter specimen quality or diagnostic accuracy.

## Introduction

Accurate diagnosis and staging of lung cancer are vital for determining treatment strategy and prognosis (1). Mediastinoscopy is widely used to visualize and obtain lymph node samples (2). However, this costly procedure, which requires surgery under general anesthesia by experienced surgeons, has recently been replaced by endobronchial ultrasound-guided transbronchial needle aspiration (EBUS-TBNA) sampling, in biopsy of mediastinal, central and hilar lesions, as well as lymph nodes within the tracheobronchial tree. EBUS-TBNA enables needle aspiration of lesions surrounding the tracheobronchial mucosa with real-time ultrasound monitoring (3,4), and is associated with lower complication rates and injury severity, higher sensitivity and specificity, and lower costs. EBUS-TBNA is also associated with improved mediastinal lymph node staging accuracy in lung cancer, and can differentiate more accurately benign from malignant, and enlarged hilar from mediastinal lymph nodes (5,6).

The puncture needle used in EBUS-TBNA contains a stylet, intended to prevent the needle cavity from being blocked by tracheal wall tissue fragments during insertion into the target lymph nodes (7). The stylet also stabilizes the needle and releases its contents in a carefully controlled manner (8,9). After the needle is introduced into a target lymph node, the stylet is removed, and suction biopsy is performed. The stylet is then reinserted before the next puncture. However, the role of the stylet has never been systematically assessed. Defects in the puncture needle with a stylet can prolong surgery and are associated with complications such as infection (10). In addition, metal particles that enter the lymph nodes after EBUS-TBNA examination may affect the structure of lymph nodes (11). However, traditional TBNA needles contain no stylet; indeed, pathological diagnosis can also be obtained using puncture suction. EBUS-TBNA without a stylet has been widely adopted in clinics. However, the influence of EBUS-TBNA without a stylet on lung cancer specimen quality has not previously been systematically assessed. In this study, we aimed to compare the quality of specimens collected by EBUS-TBNA sampling with and without a stylet in lung cancer.

## Material and Methods

### Subjects

This prospective study was designed to assess the effect of EBUS-TBNA with or without a stylet on the quality of specimens collected from patients with lung cancer, and subsequent pathological diagnostic accuracy. Between November 2013 and November 2014, we enrolled 140 consecutive patients with suspected lung cancer at our Hospital in Taizhou, Zhejiang Province, China. These patients presented with enlarged hilar or mediastinal lymph nodes. Inclusion criteria were 1) chest enhancement computed tomography (CT) scan examination with hilar and/or mediastinal lymph node enlargement of the diseased lungs, and 2) unexplained hilar, mediastinal placeholders. Exclusion criteria were severe cardiopulmonary insufficiency and blood coagulation disorders.

Prior to examination, patients were informed about the objectives, methods, and puncture procedures, as well as the possible complications and treatment outcomes. The study was approved by the Institutional Review Board of our hospital (20131230). All patients provided written informed consent.

### EBUS-TBNA procedure

Patients were requested to fast for at least 6h before surgery. Anesthesia was achieved with 2% lidocaine through thyrocricocentesis, followed by oxygen administration through a nasal catheter; electrocardiography and oxygen saturation were monitored. Surgery was performed on patients in the supine position. Patients were first subjected to conventional bronchoscopy, during which additional anesthesia (2% lidocaine) was provided. Target lymph nodes and surrounding vessels in the trachea were examined using an ultrasonic bronchoscope (BF-UC260F-OL8; Olympus Ltd, Japan) via the oral route. Ultrasound images were processed with an ultrasound image processing unit (EU-C2000; Olympus Ltd.), and the diameters of target lymph nodes were recorded. TBNA was performed only for lymph nodes exceeding 0.5 cm in diameter.

A 22G aspiration needle (NA-201SX-4022; Olympus Ltd.) was quickly thrust through the tracheal wall, until ultrasound imaging confirmed that it was located in the lesion. A negative pressure injector was then connected to the needle, and suction was performed by moving the needle back and forth 20–30 times.

The S+ group was sampled twice using a needle with a stylet, and then sampled twice using a needle without a stylet. The S– group were first sampled without a stylet twice, then with a stylet twice. All nodes were sampled four times. To prevent contamination, one needle was used for the first two punctures and another for the subsequent two, and needle cavities were rinsed with physiological saline. Surgery durations for the two punctures (with or without stylet) were calculated from needle insertion into the endoscopic biopsy channel during the first puncture to specimen collection after the last puncture. The specimens were smeared and preserved in 10% formalin. All punctures were performed by the same physician.

### Pathological evaluation and lung cancer diagnosis

Biopsy materials were immediately placed on slides with a stylet or injector, stained with hematoxylin and eosin, and air-dried; sample quality was analyzed by cytopathologists blind to experimental groups, as previously described (Table 1) (8,12). Samples scored 0–2 were considered unsuitable for diagnosis; those scored 3–6 were considered adequate for cytological diagnosis, and samples scored 7–10 were considered excellent. The two S+ and two S– samples from the same lymph node were scored as one subject.

**Table 1.** Evaluation of pathological and diagnostic results.

Criteria/Quantitative description	Score
Background blood/clot	
Large amount; great compromise in diagnosis	0
Moderate amount; diagnosis possible	1
Minimal; diagnosis	2
Amount of cellular material	
Minimal to absent; diagnosis not possible	0
Sufficient for diagnosis	1
Abundant; diagnosis possible	2
Degree of cellular degeneration	
Degeneration marked; diagnosis impossible	0
Degeneration moderate; diagnosis possible	1
Degeneration minimal; diagnosis obvious	2
Degree of cellular trauma	
Marked; diagnosis impossible	0
Moderate; diagnosis possible	1
Minimal; diagnosis obvious	2
Retention of appropriate architecture	
Minimal to absent; non-diagnostic	0
Moderate; some preservation of, e.g., follicle, papillae, acini, etc.	1
Excellent architectural display closely reflecting histology; diagnosis obvious	2

Category 1 - total score 0–2: unsuitable for diagnosis; Category 2 - total score 3–6: adequate for cytological diagnosis; Category 3 - total score 7–0: excellent for diagnosis.

Lung cancer was diagnosed where malignant cells were detected in samples, or cells suspected of being malignant in patients with clinical disease characteristics. If disease was suspected but not confirmed, patients underwent further radiological follow up (thoracoscopy, thoracotomy, or mediastinoscopy and CT-guided biopsy) after 6 months. If this follow up revealed no changes, the nodes were considered benign.

### Clinical data collection

The clinical and pathological characteristics of the patients were recorded. Lymph node locations were categorized as 2R, 4R, 4L, 7, 10R, 10L, and 11R, respectively, according to previously reported guidelines (13).

### Statistical analysis

Categorical data are reported as rate (percentage), with differences between groups analyzed by chi-square test. Continuous quantitative data are reported as means±SD, with differences between groups analyzed by *t*-test. The quality and rate of the diagnosis were assessed as previously described (9). Statistical analyses were performed with SPSS 19.0. Two-sided P<0.05 was considered to indicate statistical significance.

## Results

### Patient characteristics

Between November 2013 and November 2014, 140 patients suspected with lung cancer were enrolled. Five enrolled patients opted out before surgery, and four others did not tolerate surgery. Therefore, 131 patients (98 men and 33 women; age, 61.9±8.8 years), for total of 148 lymph nodes, were included in the final analysis (Table 2). Seventeen patients provided two mediastinal lymph node biopsies. According to the puncture results, clinical data, surgery and follow-up, the final diagnosis of 131 patients was lung cancer, including 94 cases of non-small cell lung cancer (43 cases of adenocarcinoma, 38 cases of squamous cell carcinoma, and 13 cases of unclassified carcinoma), 31 cases of small cell lung cancer and 6 cases of poorly differentiated lung cancer.

**Table 2.** Baseline characteristics of patients and lymph nodes.

Characteristic	S+ (n=74)	S– (n=57)	P value
Age, median (range) in years	61.8 (42–80)	62.1 (35–77)	0.680
Gender			
Female	17	16	0.505
Male	57	41	
Number of lymph nodes	80	68	
Lymph node size, median (cm)	1.81±0.30	1.83±0.40	0.744
Lymph node station (n)			
2R	2	1	0.036
4R	32	25	
4L	8	9	
7	26	17	
10R	5	7	
10L	3	1	
11R	4	6	
11L	0	2	

Data are reported as means±SD or numbers. S+: patients were sampled twice using a needle with a stylet, and then sampled twice using a needle without a stylet. S–: patients were first sampled without a stylet twice, then with a stylet twice. Data were analyzed with the *t*-test or chi-square test.

There was no significant difference in age, gender, and lymph node type and diameter between the two groups. All nodes were sampled four times. A total of 296 cytological specimens (148×2) were obtained for puncture with or without a stylet (Table 2). Punctures using a needle with a stylet required a mean of 14.5±0.8 min, and those using a needle without a stylet a mean of 12.7±1.1 min (P<0.001).

### Quality of the specimens collected with or without a stylet

The quality of cytological specimens acquired from lymph nodes, i.e. the scores for background blood/clot, amounts of cellular material, degree of cellular degeneration, degree of cellular trauma or retention of appropriate architecture, did not differ significantly between the sampling techniques (Table 3). The rate at which samples were considered adequate or excellent did not differ significantly between the two groups. Excellent specimens were obtained in 261/296 and 260/296 samples in the procedures with and without stylet samples, respectively (P=0.9), while the remaining 35 and 36 samples, respectively, were adequate. None of the samples was unsuitable.

**Table 3.** Quality of specimens collected with or without a stylet.

Parameter score (means±SD)	Sample		P value
	With stylet (n=296)	Without stylet (n=296)	
Background blood/Clot	1.15±0.58	1.09±0.58	0.181
Amount of cellular material	1.26±0.53	1.23±0.52	0.420
Degree of cellular degeneration	1.77±0.49	1.78±0.47	0.968
Degree of cellular trauma	1.73±0.53	1.74±0.49	0.984
Retention of appropriate architecture	1.73±0.55	1.74±0.54	0.850
Total score	7.65±0.54	7.58±0.52	0.497

### Accuracy of diagnosis made using specimens collected with or without a stylet

Correct malignant diagnosis using samples with a stylet (93.1%, 122/131) and those without a stylet (90.8%, 119/131) showed no statistically significant difference (P=0.65). There was a high degree of concordance in the determination of accuracy 91.6% (95%CI=86.8–96.4%) between the two techniques.

## Discussion

The puncture needle used in EBUS-TBNA contains a stylet. However, the role of the stylet has never been systematically assessed. This study compared the quality of specimens collected by EBUS-TBNA sampling with and without a stylet in 131 patients with suspected lung cancer.

The current study indicated that the absence of a stylet does not impair specimen quality or diagnostic efficiency in EBUS-TBNA sampling of mediastinal or hilar lymph nodes, as previously reported for similar endoscopic ultrasound-guided fine-needle aspiration procedures ([Bibr B14] [Bibr B15] 14–16). Furthermore, conventional TBNA and EBUS-TBNA have comparable diagnostic accuracy in puncture sampling of mediastinum 4R, subcarinal group, and large lymph nodes (17,18).

Diagnosis and staging accuracy of EBUS-TBNA in lung cancer is well recognized for enlarged hilar or mediastinal nodes. Therefore, the subjects assessed here were clinically considered lung cancer patients with enlarged hilar or mediastinal lymph nodes. In this study, the quality of cytological specimens acquired from the lymph nodes did not differ significantly between the samples acquired with or without a stylet. These findings confirmed the high accuracy (exceeding 90%) of lung cancer diagnosis obtained using EBUS-TBNA, both with and without a stylet, supporting the use of TBNA needles without a stylet. However, it is unclear whether similar results would be obtained for benign lesions, which requires further studies.

Interestingly, it is also worth noting that omitting the stylet from EBUS-TBNA can significantly reduce the surgical time (by about 1.8 min), potentially reducing patient discomfort level and improving physician efficiency. This finding is consistent with previous reports that omitting the stylet from EUS-guided gastroenterological procedures reduces the procedure length and risk of unintentional needle stick injuries, particularly when multiple passes are required (15,16).

However, whether the EBUS-TBNA needle should contain a stylet remains an open question. Our conclusions are limited by the scope of this study, which was conducted in a single center. In addition, the surgical staff was not blind to the endoscopic procedures, which may have biased sample collection. Furthermore, rapid on-site cytological evaluations were not performed due to shortage of staff and funds, and complication rates were not recorded. Whether the procedure conducted without a stylet is associated with a higher rate of complications remains to be investigated.

The current data, nonetheless, suggest that during EBUS-TBNA for diagnosis and staging of lung cancer, specimen collection without a stylet is easier and faster compared with collection using a stylet. The absence of a stylet in the puncture needle has no impact on specimen quality and diagnostic efficiency. These findings provide a rational basis for an alternative EBUS-TBNA method, and require further confirmation by larger multi-center studies.

## References

[1] Chao F, Zhang H (2012). PET/CT in the staging of the non-small-cell lung cancer. J Biomed Biotechnol.

[2] Hammoud ZT, Anderson RC, Meyers BF, Guthrie TJ, Roper CL, Cooper JD (1999). The current role of mediastinoscopy in the evaluation of thoracic disease. J Thorac Cardiovasc Surg.

[3] Eapen GA, Shah AM, Lei X, Jimenez CA, Morice RC, Yarmus L (2013). Complications, consequences, and practice patterns of endobronchial ultrasound-guided transbronchial needle aspiration: Results of the AQuIRE registry. Chest.

[4] Evison M, Crosbie PA, Martin J, Bishop P, Doran H, Joseph L (2014). EBUS-TBNA in elderly patients with lung cancer: safety and performance outcomes. J Thorac Oncol.

[5] Yasufuku K, Pierre A, Darling G, de Perrot M, Waddell T, Johnston M (2011). A prospective controlled trial of endobronchial ultrasound-guided transbronchial needle aspiration compared with mediastinoscopy for mediastinal lymph node staging of lung cancer. J Thorac Cardiovasc Surg.

[6] Um SW, Kim HK, Jung SH, Han J, Lee KJ, Park HY (2015). Endobronchial ultrasound versus mediastinoscopy for mediastinal nodal staging of non-small-cell lung cancer. J Thorac Oncol.

[7] Wani S, Early D, Kunkel J, Leathersich A, Hovis CE, Hollander TG (2012). Diagnostic yield of malignancy during EUS-guided FNA of solid lesions with and without a stylet: a prospective, single blind, randomized, controlled trial. Gastrointest Endosc.

[8] Maurya AK, Mehta A, Mani NS, Nijhawan VS, Batra R (2010). Comparison of aspiration *vs* non-aspiration techniques in fine-needle cytology of thyroid lesions. J Cytol.

[9] Bonifazi M, Sediari M, Ferretti M, Poidomani G, Tramacere I, Mei F (2014). The role of the pulmonologist in rapid on-site cytologic evaluation of transbronchial needle aspiration: a prospective study. Chest.

[10] Kang HJ, Hwangbo B, Lee GK, Nam BH, Lee HS, Kim MS (2014). EBUS-centred versus EUS-centred mediastinal staging in lung cancer: a randomised controlled trial. Thorax.

[11] Gounant V, Ninane V, Janson X, Colombat M, Wislez M, Grunenwald D (2011). Release of metal particles from needles used for transbronchial needle aspiration. Chest.

[12] Mair S, Dunbar F, Becker PJ, Du Plessis W (1989). Fine needle cytology-is aspiration suction necessary? A study of 100 masses in various sites. Acta Cytol.

[13] Rusch VW, Asamura H, Watanabe H, Giroux DJ, Rami-Porta R, Goldstraw P (2009). The IASLC lung cancer staging project: a proposal for a new international lymph node map in the forthcoming seventh edition of the TNM classification for lung cancer. J Thorac Oncol.

[B14] Wani S (2014). Basic techniques in endoscopic ultrasound-guided fine-needle aspiration: Role of a stylet and suction. Endosc Ultrasound.

[B15] Herth F, Becker HD, Ernst A (2004). Conventional *vs* endobronchial ultrasound-guided transbronchial needle aspiration: a randomized trial. Chest.

[16] Bellinger CR, Chatterjee AB, Chin R, Conforti J, Adair N, Haponik E (2012). Conventional and endobronchial ultrasound-guided transbronchial needle aspiration: complementary procedures. South Med J.

[17] Medford AR, Bennett JA, Free CM, Agrawal S (2010). Endobronchial ultrasound-guided transbronchial needle aspiration (EBUS-TBNA): applications in chest disease. Respirology.

[18] Wani S, Gupta N, Gaddam S, Singh V, Ulusarac O, Romanas M (2011). A comparative study of endoscopic ultrasound guided fine needle aspiration with and without a stylet. Dig Dis Sci.

